# Chemical Profile and Health-Promoting Activities of *Crataegus laciniata* (Rosaceae) Flowers

**DOI:** 10.3390/plants13010034

**Published:** 2023-12-21

**Authors:** Salvatore Mirabile, Valeria D’Angelo, Maria Paola Germanò, Shiva Pouramin Arabi, Valentina Parisi, Francesco Maria Raimondo, Emanuele Rosa

**Affiliations:** 1 Department of Chemical, Biological, Pharmaceutical and Environmental Sciences, University of Messina, Viale F. Stagno d’Alcontres 31, 98166 Messina, Italy; smirabile@unime.it (S.M.); vdangelo@unime.it (V.D.); 2 Foundation Prof. Antonio Imbesi, University of Messina, Piazza Pugliatti 1, 98122 Messina, Italy; 3 Department of Pharmacy, University of Salerno, 84084 Fisciano, Italy; spouraminarabi@unisa.it (S.P.A.); vparisi@unisa.it (V.P.); erosa@unisa.it (E.R.); 4 PLANTA/Centro autonomo di Ricerca, Documentazione e Formazione, Via Serraglio Vecchio 28, 90123 Palermo, Italy

**Keywords:** *Crataegus* Sicilian flora, hyperoside, antioxidant activity, α-amylase, α-glucosidase, tyrosinase, zebrafish embryo

## Abstract

In the present study, we focused our attention on *Crataegus laciniata* Ucria (Rosaceae), which is wild growing in western Sicily (Italy). The chemical profile of the *C. laciniata* flower’s (CLF) ethanolic (70%) extract showed the presence of both C-flavonoid and O-flavonoid derivatives. Beyond the main metabolites, like hyperoside and vitexin, there are several luteolin derivates, in addition to catechin and epicatechin dimers or trimers. Regarding the antioxidant activities, CLF showed a strong ability to scavenge DPPH and ABTS radicals and a good Fe^3+^-reducing antioxidant power. The investigation into the key enzymes in diabetes showed strong inhibition on α-amylase and α-glucosidase, whereas the skin-whitening properties are linked to inhibitory effects on tyrosinase. Moreover, we employed *Danio rerio* (zebrafish) for toxicity assessment, as it represents an ideal in vivo model due to its high correlation with humans in response to pharmaceutical and cosmetic testing. Zebrafish embryos exposed to CLF (25–100 µg/mL) showed marked depigmentation compared to phenylthiourea (PTU), in addition to a high survival percentage and the absence of malformations. In conclusion, this experimental study outlines that *C. laciniata* flowers could be a potential source of bioactive compounds for application in the pharmaceutical and cosmeceutical industries.

## 1. Introduction

*Crataegus* L. (hawthorn) is a large genus of small shrubs and trees belonging to Rosaceae family widely present in North Europe, temperate Asia, Africa and North America, including approximately 200 species. In recent years, hawthorn has been demonstrated to be an excellent source of many natural bioactive molecules, which have promising benefits for human health [1].

Many studies have shown that extracts from the fruits of hawthorn have beneficial effects on the cardiovascular system, including hypotensive activity and hypocholesterolemic and hypolipidemic effects [2]. Despite the long history of the use of the hawthorn fruits for both food and medicinal purposes, limited data are available on the active constituents and biological effects of the flowers [3].

In the present study, we focused on *Crataegus laciniata* Ucria, a species distributed through the western Mediterranean, i.e., northern Algeria, Morocco, southeastern Spain and Italy (Puglia and Sicily). *C. laciniata* has sometimes been synonymized with *C. orientalis* Pall. Ex M. Bieb. However, modern botanists are inclined to regard it as a distinct species. It is characterized by slightly lanate twigs, leaves, inflorescences, hypanthia and fruits. The subterminal leaves of flowering shoots are deeply and narrowly lobed, and they bear short petioles. The fruits are brick-red, 8–14 mm in diameter and have (1)2–3(5) pyrenes [4].

As far as we know, there are no data related to *C. laciniata* flowers. Consequently, the objective of this study was to investigate the chemical composition of a *C. laciniata* flower (CLF) ethanolic extract (70%) via HR-LC-MS analysis, as well as the antioxidant activities and in vitro health properties through the evaluation of inhibition of the key enzymes involved in diabetes (α-amylase and α-glucosidase). In addition, the effects on tyrosinase enzyme, which catalyzes the two limiting reactions of melanin biosynthesis, were also evaluated. Finally, we employed zebrafish (*Danio rerio*) embryos to evaluate both the toxicity and the whitening properties of CLF for its potential employment to counteract skin hyperpigmentation disorders. Information gained from this study can be used to support a future application of *C. laciniata* flowers as a good source of bioactive compounds.

## 2. Results

### 2.1. Total Phenolic (TPC) and Flavonoid (TFC) Content

**Figure 1:**
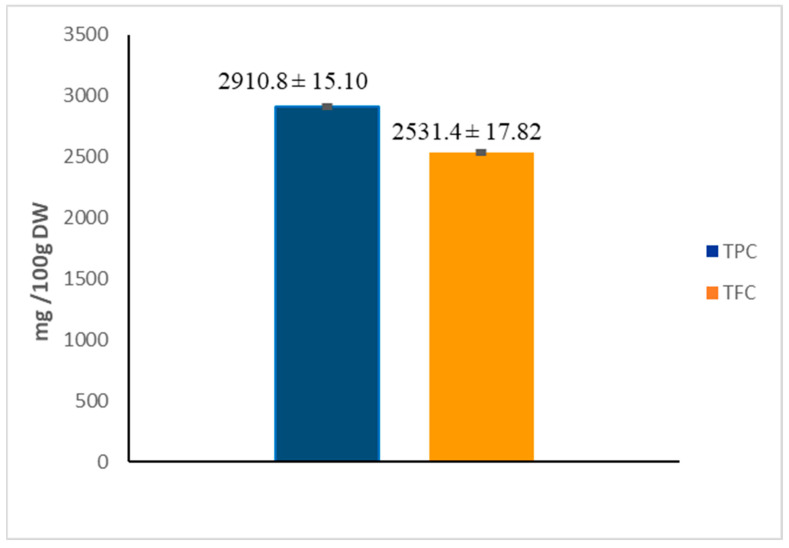
Total phenolic (TPC) and total flavonoid (TFC) contents of *C. laciniata* flower extract. Data are reported as the mean ± standard deviation (SD) of triplicate experiments (n = 3). TPC are expressed as mg gallic acid equivalents (GAE)/100 g dry weight (DW). TFC are expressed as mg rutin equivalents (RE)/100 g DW.

TPC was determined using the Folin–Ciocalteu reagent via the TFC with aluminium chloride method. The total content of polyphenols and flavonoids of hawthorn flowers is summarized in Figure 1. It was observed that CLF has a high phenolic content (2910.8 mg GAE/100 g DW), mainly represented by flavonoids (2531.4 mg RE/100 g DW).

### 2.2. Phytochemical Characterization

**Figure 2:**
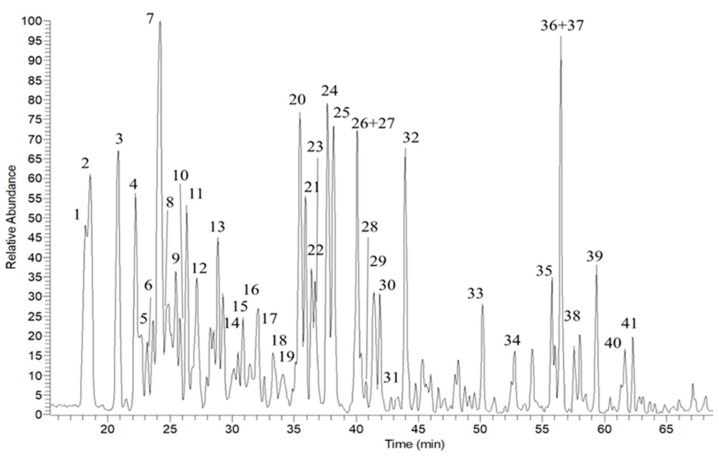
High-performance liquid chromatography–high-resolution electrospray ionization–mass spectrometry profile of CLF extract. Peaks (**1**–**41**) correspond to compounds listed in Table 1.

The untargeted analysis of CLF was effectuated in both polarities, i.e., negative- and positive-ion modes (Figure 2). Compounds **1**, **2**, **6**, **7** and **8** showed the presence of the fragment ion at *m*/*z* 161 [M-H]^−^ corresponding to dehydrated and deprotonated caffeic acid and fragment ion at *m*/*z* 191 [M-H]^−^ corresponding to deprotonated quinic acid. Several polyphenolics were identified, particularly catechin and epicatechin dimers or trimers, with compounds **10**, **11** and **15** identified as procyanidin derivates (Table 1). The most abundant polyphenols identified in the CLF extract were flavonoid glycosides, both C-flavonoid and O derivatives. Beyond the main CLF metabolites, such as hyperoside (**24**) and vitexin (**21**), there are several luteolin derivatives with different glycosylation patterns (compounds **17**, **19**, **27**, **30** and **32**). These compounds showed the fragment ion at *m*/*z* 287 [M+H]^+^ corresponding to aglycon luteolin for O-flavonoids, while for C-flavonoids, we observed a fragment ion at *m*/*z* 431 [M+H-H_2_O]^+^, corresponding to sugar’s fragmentation. Quercetin was one of the most abundant aglycons. The fragment ion at *m*/*z* 303 [M+H]^+^, corresponding to quercetin, was present in compounds **22**, **23**, **26**, **28**, **31** and **33**. Peak **26** showed the loss of *m*/*z* 162, attributed to hexose units; **22** and **23** showed the double loss of *m*/*z* 162 and 146, corresponding to losses of hexose and deoxyhexose, respectively. In contrast, **31** and **33** showed the fragmentation pattern of C-hexoside with fragment ion at *m*/*z* 445 [M+H-C_2_H_3_O-H_2_O]^+^. The extract of CLF is very rich in anthocyanidins; in particular, compounds **16**, **18** and **25** show the same fragment ion at *m*/*z* 303 [M]^+^, corresponding to a delphinidin aglycon. Finally, several polyunsaturated fatty acids and hydroxylated polyunsaturated fatty acids were observed at the end of the chromatogram. The identified metabolites in CLF are listed in Table 1.

### 2.3. Antioxidant Activity

Considering the phytochemical composition of the *C. laciniata* flower extract, different in vitro tests were performed to assess its antioxidant potential. Our results are reported in Table 2 and show a good antioxidant activity in DPPH and FRAP assays. Interestingly, in the TEAC test, CLF exhibited a strong ability to scavenge the ABTS radical, which provided results comparable to Trolox, used as positive control.

### 2.4. α-Amylase and α-Glucosidase Inhibitory Activity

The inhibitory effects on α-amylase and α-glucosidase enzymes were evaluated. Our results show that CLF inhibited both the enzymes, even if the effects on α-glucosidase were higher than α-amylase. Interestingly, CLF was demonstrated to be 100-fold more potent than the reference compound acarbose (4.56 vs. 436.47 µg/mL). The IC_50_ values of CLF are reported in Table 3 and compared to those of acarbose.

### 2.5. Inhibitory Effects on Tyrosinase Enzyme

*C. laciniata* extract was investigated regarding tyrosinase, and our results shown in Table 4 were compared to kojic acid, used as a positive control. The inhibitory effects of CLF are already evident in the first step of the reaction, with a lower IC_50_ value for monophenolase than diphenolase activity (67.47 vs. 103.61 µg/mL).

### 2.6. Inhibitory Effects on Melanogenesis in Zebrafish Embryos

**Figure 3:**
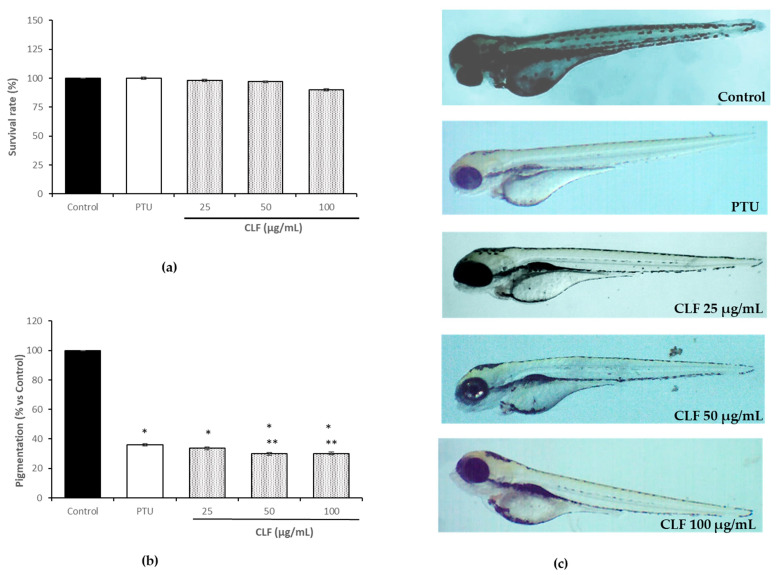
(**a**) The survival rate of zebrafish embryos after 72 h of treatment with PTU and *C. laciniata* flower extract (25–100 µg/mL). (**b**) Effects of CLF on the pigmentation of zebrafish embryos after treatment. Pigmentation was expressed as % vs. control. * *p* < 0.01, vs. control. ** *p* < 0.05, vs. PTU (**c**) Images of zebrafish embryos at 72 hpf (magnification 20×) were captured using a stereomicroscope (SMZ-171 Series, Motic, San Antonio, TX, USA) equipped with a digital camera (MotiCam^®^ 5 plus, Motic, San Antonio, TX, USA).

In this study, a toxicological evaluation of zebrafish’s early-stage development was performed before evaluating the anti-melanogenic effects of CLF. In addition to a good survival percentage (90%), the absence of morphological abnormality in treated concentrations of CLF (25–100 µg/mL) until the end of the testing period (72 h post-fertilization, hpf) was observed (Figure 3, Panel a). The same treatment allowed us to evaluate the anti-melanogenic effects on zebrafish. After a visual inspection of the embryos under a stereomicroscope, images were captured and processed to quantify the whitening effects induced via CLF treatment with respect to the control (100% pigmentation) (Figure 3, Panel b and c). Overall, embryo pigmentation was significantly reduced in a dose-dependent manner after treatment with 25 and 50 µg (33.64% and 29.87% vs. control, respectively); however, the highest concentration (100 µg/mL) did not result in a further increase in depigmentation (30.19% vs. control). In general, it was found that treatment with CLF (25–50 µg) was slightly more effective than that with 1-Phenyl-2-thiourea (PTU), used as the reference standard (30 µg/mL, 36.00%).

## 3. Discussion

*Crataegus* species (hawthorn) have been used traditionally since ancient times. Hawthorn contains a variety of flavonoids and anthocyanins that appear to be primarily responsible for the cardiac action of the plant [6]. Our research was focused on *C. laciniata*, a species growing in Sicily (Nebrodi and Madonie mountains) described for the first time by Bernardino ab Ucria [4].

Firstly, we quantified the phenolic and flavonoid contents of a flower ethanolic extract, which proved to be particularly rich in flavonoids (2531.4 mg/100 g DW). Our results are similar to those reported for samples of *Crataegus oxyacantha* L. flowers from Algeria with a TPC average of 2759.0 mg GAE/100 g DW. However, data regarding TFC (660.7 mg RE/100 g DW) was lower than for our samples of *C. laciniata* [3]. Among the limited data regarding the flavonoid contents of the flowers of different *Crataegus* species, Edwards et al. reported lower values ranging from 317.8 to 1710 mg/100 g DW [6]. Therefore, when compared with the literature, variability can be attributed to the different locations and species.

Subsequently, through LC-MS/MS analysis, the phytochemical composition was determined. The phytochemical profile of CLF was rich in polyphenols. The most abundant polyphenols identified in CLF were flavonoid derivatives [7,8]. In addition to hyperoside and vitexin as the main metabolites, luteolin glycosides were detected. These results are in accordance with a previous study of *Crataegus oxyacantha* flowers that were rich in flavones like vitexin, as well as flavonols like rutin and hyperoside [3]. CLF was also found to be very rich in anthocyanidins, containing delphinidin aglycon [9]. Moreover, catechin and epicatechin polymers that corresponded to procyanidin derivates were identified. A similar procyanidin profile was also reported in flowers of two varieties of *C. azarolus* (*C. azarolus* L. var. *aronia* Batt. and *C. azarolus* L. var. *eu-azarolus* Maire) growing in Tunisia [10].

Oxidative stress generates excessive reactive oxygen species, which cause damage to cells, accelerating age-related dysfunctions and producing chronic diseases. To evaluate the antioxidant activity of our extract, we used DPPH, TEAC and FRAP tests. The results indicate that CLF has a good scavenger activity against DPPH (IC_50_ = 165.25 µg/mL). Interestingly, regarding TEAC assay, CLF possesses an IC_50_ value of 20.13 µg/mL, comparable to Trolox (IC_50_ = 13.08 µg/mL), due to the synergistic effects of its mixture of polyphenolic compounds. The same extract also showed a good antioxidant activity in the FRAP test (75.61 TE/g extract), supporting the reducing ability of its compounds.

The health-promoting activities of CLF were also investigated in vitro for two enzymes (α-amylase and α-glucosidase). The inhibition of α-amylase and α-glucosidase have seemed to be an important therapeutic target for the management of diabetes. Post-prandial hyperglycemia is modulated via the inhibition of these enzymes, which results in the delay of the carbohydrate digestion and glucose absorption. As depicted in Table 3, CLF displayed dual α-amylase (IC_50_ = 517.41 µg/mL) and α-glucosidase (IC_50_ = 4.56 µg/mL) inhibitory activity. In particular, the strong effect on this latter enzyme is comparable to those obtained with Irish seaweed extracts, reaching IC_50_ values below 2 µg/mL [11]. The high antihyperglycemic capacity of CLF could be related to its phytochemical composition, rich in glycosides such as hyperoside and vitexin. Indeed, recent studies reported that the α-amylase activity decreased in the presence of hyperoside in a competitive manner [12]. On the other hand, vitexin exhibited a potent inhibitory ability on α-glucosidase, with an uncompetitive mechanism of action [13].

Regarding the anti-melanogenic activity in vitro and in vivo, tests were performed on tyrosinase enzyme (TYR) and zebrafish embryos, respectively. TYR has been recognized as a key target for the screening of novel bioactive agents for dermatological disorders based on melanin accumulation. TYR catalyzes the two limiting reactions of melanin biosynthesis: the hydroxylation of L-tyrosine to L-DOPA (monophenolase activity), which is oxidized to form dopaquinone (diphenolase activity) [14].

The well-known TYR inhibitors, such as kojic acid, could be responsible for adverse effects occurring because of long-term application [15]. Due to these safety concerns, many medicinal plants have been screened using an in vitro TYR inhibition assay to search novel phytocomplexes with skin-whitening properties. Among different *Crataegus* species, it was previously reported that extracts from *C. azarolus* L. aerial parts and *C. pinnatifida* Bunge seeds reduced the melanin content in B16F10 cells by inhibiting the TYR activity [16,17]. On this basis, we investigated the effects of *C. laciniata* flowers on TYR. The results revealed that CLF inhibits both the monophenolase and the diphenolase activity at low concentrations (IC_50_ = 67.47 and 103.61 µg/mL, respectively). Considering that hyperoside and vitexin, the main metabolites identified in CLF, are effective TYR inhibitors, their involvement in anti-melanogenic activity cannot be excluded [18,19].

The zebrafish embryo is an accepted model for biochemical studies due to its high physiological and genetic similarity to mammals. For this reason, zebrafish embryo assays emerge as replacement approaches for animal experiments [20]. Zebrafish have several advantages, including small size and easy handling, as well as good absorption of test samples through the skin in the early stage. Moreover, the optically transparent embryogenesis allows us to observe the pigmentation process [21]. Accordingly, in this study, CLF showed strong whitening effects on the pigmentation of early-stage embryos without affecting their development and survival after 48 h of exposure. In similar experimental conditions, dibenzofuran compounds isolated from *C. pycnoloba* Boiss. and Heldr. aerial parts reduced embryo melanogenesis at 10 µg/mL [20]. Interestingly, the rich polyphenolic phytocomplex of CLF induced embryo depigmentation at an already low dosage (25 µg/mL), being slightly more effective than the standard PTU.

## 4. Materials and Methods

### 4.1. Sample Preparation and Extraction

*C. laciniata* flowers were collected from wild-growing plants in the locality Canna (Madonie, Sicily) at 1610 m (a.s.l.) during June 2022. A voucher specimen identified by Prof. F.M. Raimondo was deposited in PAL-Gr. For the experiments, flowers (5 g) were air-dried in the shade and extracted with 70% (*v*/*v*) ethanol (50 mL) via stirring on a plate for 10 min. Subsequently, the mixture was ultrasonicated for 30 min at 25 °C. The obtained extract was concentrated until achieving dryness (yield 12.76%).

### 4.2. Chemicals and Reagents

All chemicals and reagents, solvents, α-amylase (EC 3.2.1.1), α-glucosidase (EC 3.2.1.20) and mushroom tyrosinase (EC 1.14.18.1) were purchased from Merck (Milan, Italy).

### 4.3. Determination of Total Phenolic Content (TPC)

TPC was determined using the Folin–Ciocalteu reagent with small modifications [22]: 100 µL of sample solution (1 mg/mL) was initially diluted with 2000 µL of distilled water and subsequently mixed with 200 µL of the Folin–Ciocalteu reagent. After 3 min, 1000 µL of Na_2_CO_3_ (15%) was added. The reaction mixture was then incubated in the dark at room temperature for 1 h. At the end, the absorbance of the sample was measured at 765 nm using a spectrophotometer (UV-Spectrophotometer Cary 60, Agilent Technology, Milan, Italy). TPC was expressed in mg gallic acid equivalents (GAE) per 100 g of dry weight (DW) using a calibration curve of this compound.

### 4.4. Determination of Total Flavonoid Content (TFC)

TFC was determined according to the method of Xiong et al., albeit with some modifications [23]: 100 µL of extract (1 mg/mL) was initially diluted with 400 µL of distilled water and then mixed with 30 µL of NaNO_2_ (5%). After 5 min, 30 µL of AlCl_3_ (10%) were combined with the reaction mixture, and 6 min later, 200 µL of NaOH (1 M) and 240 µL of distilled water were finally added. The absorbance of the reaction mixture was then measured spectrophotometrically at 510 nm. The results were expressed as mg rutin equivalents (RE) per 100 g DW.

### 4.5. LC-MS/MS Qualitative Analysis

The separation system adopted was an Accela (Thermo Fisher Scientific, Milan, Italy) HPLC interfaced through an ESI source to a linear ion trap coupled to a high-resolution mass analyzer (LTQ-Orbitrap XL, Thermo Fisher Scientific, Milan, Italy) operating in negative- and positive-ion modes. HRESIMS data were obtained in both the positive- and negative-ion modes [24]. The MS data were first acquired in the full-mass and data-dependent-scan modes; then, tandem MS experiments were performed to identify the specialized metabolites. A C18 column (Luna C18 150 × 2.0 mm, 3 µm) (Phenomenex®, Castel Maggiore, Bologna, Italy) and a binary mobile phase composed of eluent A (ultrapure water–formic acid 0.1% *v*/*v*) and eluent B (ultrapure acetonitrile–formic acid 0.1% *v*/*v*) were used. The separation conditions ranged from 5% to 60% of B in 35 min and then to 100% in 15 min. The flow rate was set to 0.200 mL/min, and the injection volume was 10.0 µL [25].

### 4.6. Determination of Antioxidant Activity

The antioxidant activities of *C. laciniata* flower extracts were determined using three assays: 1,1-diphenyl- 2-picrylhydrazyl (DPPH) and 2,2′-azino-bis(3-ethylbenzothiazoline-6-sulfonic acid) (ABTS) radical scavenging activity and Fe^3+^-reducing antioxidant power (FRAP). DPPH scavenging activity was measured using the method of Xiong et al., albeit with minor modifications [23]. In brief, 0.5 mL of sample (50–250 µg/mL) was added to 3.0 mL of DPPH methanolic solution (0.1 mM). After shaking, the mixture was stored in the dark for 30 min at room temperature, and, finally, the absorbance was measured at 517 nm. Ascorbic acid served as the positive control. The percentage of DPPH scavenging activity was calculated using the following formula:

Inhibition(%)=(C−S)/C∗100

where C = the absorbance of the control, and S = the absorbance of the extract.

Data were expressed as the concentration for 50% scavenging activity of DPPH radical (IC_50_).

The free radical scavenging activities of extracts were also determined via the TEAC test using ABTS radical following the method reported by Wang et al. [26]. Samples were tested ranging from 12.5 to 125 µg/mL. Trolox was used as a positive control. The percentage of ABTS free radical scavenging activity was calculated using the following formula:

Inhibition(%)=(C−S)/C∗100

Data were expressed in terms of the decrease in ABTS scavenging activity by 50%.

The FRAP test was evaluated following the method reported by Uysal et al., employing the following mixture as a reagent [27]: 2,4,6-tri(2-pyridyl)-s-triazine, acetate buffer and FeCl_3_ 6H_2_O. Samples were tested ranging from 12.5 to 125 µg/mL. The results were reported as mg Trolox equivalents (mg TE/g extract).

### 4.7. α-Amylase Inhibitory Assay

The α-amylase inhibition assay was performed using the 3,5-dinitrosalicylic acid (DNSA) method, albeit with minor modifications [28]. A mixture of 40 µL of *C. laciniata* flower extract in DMSO (0.25–1.0 mg/mL), 160 µL distilled water and 200 µL α-amylase enzyme (4 U/mL dissolved in buffer Na_2_HPO_4_/NaH_2_PO_4_ 0.02 M containing NaCl 0.006 M, pH 6.9) was incubated at 30 °C for 10 min. Then, 400 µL of potato starch (0.5%, *w*/*v*) were added to the mixture and re-incubated via the method previously described.

At the end, 200 μL of mixture was removed, added into a separate tube containing 100 µL of DNSA reagent solution (96 mM 3,5-dinitrosalicylic acid, 5.31 M sodium potassium tartrate in 2 M NaOH) and placed into a water bath at 90 °C. After 15 min, this mixture was diluted with 900 µL distilled water, and the absorbance was measured at 540 nm using a UV-Visible spectrophotometer. The control with 100% enzyme activity was prepared by replacing the extract with DMSO (40 μL). A blank was similarly prepared by replacing the enzyme solution with distilled water to allow absorbance produced by the extract. Acarbose (10–100 µg/mL) was used as a positive control.

The α-amylase inhibitory activity was calculated using the following equation:

Inhibition(%)=(C−S)/C∗100

where C = the absorbance of the control, and S = the absorbance of the extract—blank

The results are reported as the concentration inhibiting 50% of the enzymatic activity (IC_50_).

### 4.8. α-Glucosidase Inhibitory Assay

The α-glucosidase inhibition assay was performed according to the procedure described by Milella et al., albeit with minor modifications [29]. The reaction mixture was prepared with 650 µL of phosphate buffer (0.1 M, pH 6.8), 200 µL of the test sample (1–100 µg/mL) and 100 µL of enzyme solution (0.4 U/mL in buffer) and incubated at 37 °C for 15 min. Then, 300 μL of 4-nitrophenyl α-D-glucopyranoside (2.5 mM in buffer) was added to the mixture and re-incubated via the method previously described. Absorbance was measured at 405 nm. The control with 100% enzyme activity was prepared by replacing the extract with buffer (200 μL). A blank was similarly prepared by replacing the enzyme solution with buffer to determine the absorbance produced by the extract. Acarbose (10–1000 µg/mL) was used as a positive control. The inhibition percentage was calculated via the following equation:

Inhibition(%)=(C−S)/C∗100

where C = the absorbance of the control, and S = the absorbance of the extract—blank.

The results are reported as IC_50_.

### 4.9. Tyrosinase Enzyme Inhibitory Assay

An in vitro assay was performed according to the method of Mirabile et al. [30]. The tyrosinase inhibitory effects of *C. lacianiata* flower extracts were evaluated based on both the monophenolase and diphenolase activities of a tyrosinase from *Agaricus bisporus*. The tests were conducted as follows: aliquots (50 µL) of extract (50–250 µg/mL) were mixed with 500 µL of substrate, L-DOPA or L-tyrosine (1.25 mM) and 900 µL of phosphate buffer (50 mM, pH 6.8). After 10 min of incubation at 25 °C, 50 µL of enzyme (333 U/mL) was added to the reaction mixture. To evaluate the inhibitory effects, absorbance was recorded at 475 nm for up to 40 min to evaluate the monophenolase activity, as well as after just 60 s for the diphenolase activity. DMSO and kojic acid (1–25 μg/mL) were used as negative and positive controls.

The inhibitory effects on tyrosinase enzyme activity were calculated using the following equation:

Inhibition(%)=(C−S)/C∗100

where C = tyrosinase + substrate (L-DOPA or L-tyrosine), and S = tyrosinase + substrate (L-DOPA or L-tyrosine) + sample.

The results are reported as the concentration inhibiting 50% of the enzymatic activity (IC_50_).

### 4.10. Zebrafsh Embryo Maintenance and Treatment

Adult zebrafish specimens (male and female) were maintained in a temperature-controlled aquarium (28.5 °C) with light-dark cycles and regularly fed with *Artemia salina* larvae [31]. After natural deposition, the laid eggs were collected and incubated at 28.5 °C. After 24 h, all the eggs were observed under a stereomicroscope (SMZ-171 Series, Motic, Hong Kong, China) to select the fertilized ones. The vitality test and whitening effect were evaluated in vivo on zebrafish embryos according to the following method [32]. The eggs at 24 hpf (hours post-fertilization) wee manually stripped of the chorion, and the embryos were distributed into 96-well plates (1 embryo per well), randomly divided into four experimental groups (20 embryos per three replicates for each group) and subjected to treatment with CLF (25, 50 and 100 µg/mL), before being solubilized in DMSO (0.2%). Control embryos were incubated with embryo water (negative control). After 48 h of incubation at 28 °C (72 hpf), the effects of the treatment were observed using a stereomicroscope (SMZ-171 Series, Motic) equipped with a digital camera (Moticam^®^ 5 plus) for image acquisition. The abnormal phenotypes and mortality of each treated group were documented. Regarding the whitening effects, the Pillow library for the Python programming language was used to process the acquired images of the CLF treatment (25–100 μg/mL), PTU (30 μg/mL) and controls. The images were converted to gray scale, and the pixel measurements analyzer program was used to count the area of the zebrafish image pigmentation. The quantification of pigmentation was expressed as a percentage change compared to the control group, which was considered to be 100%. All experiments were performed in compliance with the European Directive 2010/63/EU and following the ethical guidelines described by the National Institute of Health Guide for the Care and Use of Laboratory Animals.

### 4.11. Statistical Analysis

The statistical significance was evaluated via one-way analysis of variance (ANOVA). Data were considered statistically significant for *p* < 0.05 and *p* < 0.01.

## 5. Conclusions

In conclusion, the aim of this study was the investigation of *Crataegus laciniata* flower (CLF) extract. The obtained results outline that CLF is a rich source of bioactive compounds, which can be used for the treatment of metabolic disorders, as well as skin hyperpigmentation. In addition, its favourable safety profile observed during in vivo experiments might promote future applications in the pharmaceutical and cosmeceutical fields.

## Figures and Tables

**Table 1:** Secondary metabolites identified in CLF extract via LC-MS/MS analysis (t_r_ = retention time; MSI = Metabolomics Standards Initiative).

Peaks	t_r_ (min)	[M+H]^+^	[M-H]^−^	Fragmentation ^a^	Compound	Formula	Error (ppm)	MSI Level ^a^
**1**	18.16	517.1550		355	Caffeoylquinic acid-*O*-hexoside	C_22_H_28_O_14_	−0.34	2
**2**	18.52	517.1550		355	Caffeoylquinic acid-O-hexoside isomer	C_22_H_28_O_14_	−0.34	2
**3**	20.77	449.1057 ^b^		287	Cyanidin-O-hexoside	C_21_H_21_O11	−4.67	1
**4**	22.50		337.0928	191;161	Coumaraylquinic acid	C_16_H_18_O_8_	2.98	2
**5**	22.94		325.0945	161	Coumaric acid O-hexoside	C_15_H_18_O_8_	8.32	2
**6**	23.22		515.1176	323	Dicaffeoylquinic acid	C_25_H_24_O_12_	1.55	2
**7**	24.11		353.0851	161	Caffeoylquinic acid	C_16_H_18_O_9_	−4.55	2
**8**	25.33		515.138	355;161;191	Caffeoylquinc acid O-hexoside	C_22_H_28_O_14_	−2.97	2
**9**	25.43	387.2004		369;207	Roseoside	C_19_H_30_O_8_	−2.44	2
**10**	26.29		577.1372	425;289	Procyanidin dimer	C_30_H_26_O_12_	5.54	2
**11**	26.91		577.1372	425;290	Procyanidin dimer isomer	C_30_H_26_O_12_	5.54	2
**12**	27.14	611.1580		433	Rutin	C_27_H_30_O_16_	−4.35	1
**13**	28.44	291.0863		273;165;151;123	Catechin	C_15_H_14_O_6_	−0.05	2
**14**	31.06	369.1180		145;117	Feruloylquinic acid	C_17_H_20_O_9_	−0.03	2
**15**	31.63		865.1974	695;577;407;287	Procyanidin trimer	C_45_H_38_O_18_	0.00	2
**16**	32.23	627.1565 ^b^		465;303	Delphinidin-O-dihexoside	C_27_H_31_O_17_	1.59	2
**17**	32.52	449.1078		431,416,383	Luteolin-C-hexoside	C_21_H_20_O_11_	−0.08	2
**18**	32.7	627.1565 ^b^		465;303	Delphinidin-O-dihexoside isomer	C_27_H_31_O_17_	1.59	2
**19**	33.33	449.1078		431,416,383	Luteolin-C-hexoside isomer	C_21_H_20_O_11_	−0.08	2
**20**	35.4	565.1552		433;415	Apigenin-C-hexoside-O-pentoside	C_26_H_28_O_14_	0.03	2
**21**	35.8	433.1116		415	Vitexin	C_21_H_20_O_10_	−3.05	1
**22**	36.33	611.1577		465;303	Quercetin-O-hexoside-rhamnoside	C_27_H_30_O_16_	−4.84	2
**23**	36.57	611.1577		465;303	Quercetin-O-hexoside-rhamnoside isomer	C_27_H_30_O_16_	−4.84	2
**24**	37.57	465.1010		303	Hyperoside	C_21_H_20_O_12_	−3.77	1
**25**	38.09		465.1000	301	Delphinidin-O-hexoside	C_21_H_21_O_12_	−5.80	2
**26**	40.03	479.1163		317	Methoxyquercetin-O-hexoside	C_22_H_22_O_12_	−4.38	2
**27**	40.26	449.1078		287	Luteolin-O-hexoside	C_21_H_20_O_11_	−0.08	2
**28**	40.33	625.1732		479;317	Methoxyquercetin-O-hexoside-rhamnoside	C_28_H_35_O_16_	−4.95	2
**29**	41.44	449.1057		287	Luteolin-O-hexoside	C_21_H_20_O_11_	−4.76	2
**30**	41.46	449.1078		287	Luteolin-O-hexoside	C_21_H_20_O_11_	−0.08	2
**31**	43.4	507.2233		463;445;343;301	Acetylquercetin-C-hexoside	C_23_H_22_O_13_	−0.08	2
**32**	45.37	535.1069		287	Luteolin-O-malonylhexoside	C_24_H_22_O_14_	−2.42	2
**33**	50.12	507.2233		463;445	Acetylquercetin-C-hexoside isomer	C_23_H_22_O_13_	−0.08	2
**34**	54.16		329.2310	311;313;293;275	Trihydroxyoctadecenoic acid	C_18_H_34_O_5_	−3.64	2
**35**	55.7		331.2465	313;295;277	Trihydroxyoctadecanoic acid	C_18_H_36_O_5_	−4.22	2
**36**	56.04		293.2104	275	Hydroxyoctadecatrienoic acid	C_18_H_30_O_3_	−2.38	2
**37**	56.43		311.2212	293;275	Dihydroxyoctadecatrenoic acid	C_18_H_32_O_4_	−1.28	2
**38**	57.5	333.2617		315;297;279	Trihydroxyoctadecanoic acid isomer	C_18_H_36_O_5_	−5.40	2
**39**	59.31	289.2367		271;253;235	Dihydroxyphenylnonanoic acid	C_16_H_32_O_4_	−2.07	2
**40**	61.31	309.2074		291;273	Dihydroxyoctadecatrenoic acid isomer	C_18_H_28_O_4_	4.54	2
**41**	62.28	309.2074		291;274	Dihydroxyoctadecatrenoic acid isomer II	C_18_H_28_O_4_	4.54	2

^a^ Sumner et al., 2007 [5]. ^b^ m/z values are expressed as [M]^+^.

**Table 2:** Antioxidant activity of *C. laciniata* flower (CLF) extract.

	DPPH (IC_50_ µg/mL)	TEAC (IC_50_ µg/mL)	FRAP (TE/g Extract)
CLF	165.25 ± 0.15	20.13 ± 0.09	75.61 ± 0.025
Ascorbic acid	16.95 ± 1.20	----	----
Trolox	----	13.08 ± 0.02	----

Data are reported as the mean ± standard deviation (SD) of triplicate experiments (n = 3). IC_50_ = concentration giving 50% of the activity; TE = Trolox equivalents.

**Table 3:** α-amylase and α-glucosidase inhibition of *C. laciniata* flower (CLF) extract.

	α-Amylase (IC_50_ µg/mL)	α-Glucosidase (IC_50_ µg/mL)
CLF	517.41 ± 36.86	4.56 ± 0.13
Acarbose	29.07 ± 1.9	436.47 ± 58.82

Data are reported as the mean ± standard deviation (SD) of triplicate experiments (n = 3). IC_50_ = concentration inhibiting 50% of the enzyme activity.

**Table 4:** Tyrosinase inhibition of *C. laciniata* flower (CLF) extract.

	Monophenolase (IC_50_ µg/mL)	Diphenolase (IC_50_ µg/mL)
CLF	67.47 ± 3.86	103.61 ± 5.46
Kojic acid	9.18 ± 1.24	2.52 ± 0.81

Data are reported as the mean ± standard deviation (SD) of triplicate experiments (n = 3). IC_50_ = concentration inhibiting 50% of the enzyme activity.

## Data Availability

Data are contained within this article.
